# Role of long non‐coding RNA MIAT in proliferation, apoptosis and migration of lens epithelial cells: a clinical and *in vitro* study

**DOI:** 10.1111/jcmm.12755

**Published:** 2016-01-28

**Authors:** Yi Shen, Ling‐Feng Dong, Rong‐Mei Zhou, Jin Yao, Yu‐Chen Song, Hong Yang, Qin Jiang, Biao Yan

**Affiliations:** ^1^ Eye HospitalNanjing Medical UniversityNanjingChina; ^2^ The Fourth School of Clinical MedicineNanjing Medical UniversityNanjingChina

**Keywords:** long non‐coding RNA, cataract, circulating RNA, cell viability, cell migration

## Abstract

Age‐related cataract is among the most common chronic disorders of ageing and is the world's leading blinding disorder. Long non‐coding RNAs play important roles in several biological processes and complicated diseases. However, the role of lncRNAs in the setting of cataract is still unknown. Here, we extracted total RNAs from the transparent and age‐matched cataractous human lenses, and determined lncRNA expression profiles using microarray analysis. We found that 38 lncRNAs were differentially expressed between transparent and cataractous lenses. 17 of 20 differentially expressed lncRNAs were further verified by quantitative RT‐PCRs. One top abundant lncRNA, MIAT, was specifically up‐regulated both in the plasma fraction of whole blood and aqueous humor of cataract patients. MIAT knockdown could affect the proliferation, apoptosis and migration of Human lens epithelial cells (HLECs) upon oxidative stress. Posterior capsule opacification (PCO) is a common complication of cataract surgery, which is associated with abnormal production of inflammatory factors. MIAT knockdown could repress tumour necrosis factor‐α‐induced abnormal proliferation and migration of HLECs, suggesting a potential role of MIAT in PCO‐related pathological process. Moreover, we found that MIAT acted as a ceRNA, and formed a feedback loop with Akt and miR‐150‐5p to regulate HLEC function. Collectively, this study provides a novel insight into the pathogenesis of age‐related cataract.

## Introduction

The lens is composed of lens epithelium, and is usually a transparent and biconvex structure. It deteriorates with age, ultraviolet radiation, oxidative stress and other toxic factors, eventually resulting in the formation of cataract 1, 2. Many morphological and functional changes take place in the process of cataract formation for lens cells, including increased proteolysis, altered cell cycle, DNA damage and the change in growth and differentiation of lens epithelial cells (LECs) 3.

Accumulating evidence reveals that gene expression in the lens epithelium is significantly altered during cataract formation. For instance, metallothionein IIA, osteonectin and adhesion‐related kinase are up‐regulated in cataractous lenses relative to transparent lenses 4, 5, 6. By contrast, many ribosomal proteins and protein phosphatase 2A are down‐regulated in cataractous lenses relative to transparent lenses 7, 8. Metallothionein IIA is involved in metal binding and detoxification. Osteonectin, a calcium‐binding protein, is a key regulator of cell growth. Reduced expression of ribosomal proteins causes decreased protein synthesis, a pathological process involved in cataract formation 2. Although these changes in gene expression are informative, further gene identifications are still required to elucidate the molecular mechanism of cataract formation.

LncRNAs are identified as non‐coding RNA molecules greater than 200 nucleotides in length 9, 10. They regulate gene expression in diverse biological processes. Aberrant lncRNA expression is associated with several human disorders, including cancers, cardiovascular diseases and neurological diseases 11, 12. Recently, some lncRNAs are found to play a critical role in eye development and diseases 13, 14, 15. However, it is still unknown about the role of lncRNA in human lenses.

Here, we compared the difference of lncRNA expression between the transparent and cataractous human lenses. We found that LncRNA‐MIAT was significantly up‐regulated in cataractous lenses. *In vitro* studies revealed that MIAT knockdown could affect proliferation, apoptosis and migration of LECs, implying a potential role of MIAT in cataract formation.

## Materials and methods

### Clinical sample collection and inclusion criteria

This clinical study was approved by the ethics committees of Nanjing Medical University (Nanjing, China). The surgical specimens were handled in accordance with the Declaration of Helsinki. Written informed consent for specimen collection and subsequent analysis was obtained from every enrolled participant.

The lens samples for lncRNA microarray were collected from postmortem eyes (nine donors, age range was 54–69 years, free of ocular diseases) and age‐related cataract patients (nine patients, age range was 49–70 years, free of other ocular diseases). All lens samples were obtained by intact continuous curvilinear capsulorhexis, with care taken to avoid vascular contact or damage to the iris or other intraocular structures. As a result of one lens did not contain sufficient RNA for microarray analysis, three transparent or cataractous lenses were pooled together as a biological repeat to obtain enough RNA. The degree of lenticular opacification was determined using the Lens Opacities Classification System III.

Lenses from postmortem eyes were obtained from the Eye Bank in Nanjing, China, within 24 hrs after death. The lenticular opacification of these lenses ranged from grade 1 to 2, and these samples were used as the transparent samples. The cataractous lens samples had lenticular opacification ranging from grade 4 to 6. These lenses were acquired during surgeries performed on age‐related cataract patients after informed consent of each patient was obtained (Table S1). No statistical significance of age was detected between the transparent and cataractous lenses (*P* > 0.05, Independent sample *t*‐test).

Approximately 100 μl of aqueous humor (AH) was collected from cataract, PVR, glaucoma, or traumatic (free of other ocular diseases) patients using a 30‐gauge needle inserted through the peripheral cornea. The needle did not contact any iris or lens tissue during the sample collection. Aqueous humor samples were centrifuged to remove cellular components at 300 × g for 15 min., and its aqueous phase was gently collected. The whole blood was collected from cataract patients, PVR patients, glaucoma patients or healthy controls (free of other ocular disease). The whole blood was centrifuged at 1000 × g for 10 min. to obtain the plasma and cellular fraction.

### RNA extraction and lncRNA microarray analysis

Total RNAs were extracted using the TRIzol reagent (Invitrogen, Carlsbad, CA, USA). Reverse transcription polymerase chain reaction (RT‐PCR) was performed with the one‐step reverse transcription‐polymerase chain reaction system (Takara, Dalian, China) according to the manufacturer's protocol. LncRNA microarray analysis, including labelling, hybridization, scanning, normalization and data analysis, was performed by Shanghai OE Biotech. Co., Ltd. (Shanghai, China) using the Agilent Human Gene Expression (8 × 60K, Design ID: 039494).

### Cell culture

SV40 T‐antigen‐transformed human LEC line (SRA01/04 cell) was cultured in DMEM (Gibco, Carlsbad, CA, USA) supplemented with 10% heat‐inactivated foetal bovine serum (Hyclone, Rockford, IL, USA), 100 U/ml penicillin and 100 U/ml streptomycin 16. Cells were incubated in a humidified 37°C incubator containing 5% CO_2_. Unless otherwise indicated, these cells were grown to 70–80% confluence and then serum‐starved overnight prior to treatment.

### siRNA transfection

MIAT siRNA and scrambled siRNA were purchased from Shanghai GenePharma Co., Ltd (Shanghai, China). siRNA was transfected using lipofectamine 2000 transfection reagent (Invitrogen) according to the manufacturer's protocol. A total of 5 × 105 cells in 2 ml of medium were seeded in 6‐well plates. siRNA (10 nM) was then gently introduced into the cells by mixing with the required amount of transfection reagent.

### Cell migration assay

HLECs were grown to a monolayer and then placed in serum‐free medium for 24 hrs. After medium was removed, a straight line scratch was scraped across cell layer with a pipette tip, and then rinsed with PBS to remove suspended cells. These cells were transfected with MIAT siRNA, scrambled siRNA or left untreated, then treated with tumour necrosis factor (TNF)‐α (10 ng/ml) for indicated time. The scratch gap was recorded and photographed using an Olympus (DB80; Tokyo, Japan) microscope.

### Calcein‐AM and propidium iodide double staining

Calcein‐AM and propidium iodide (PI) double staining was used to discriminate live and dead cells. After specific treatment, HLECs were fixed with 70% ethanol for 30 min., and then these cells were stained with Calcein‐AM solution (10 μmol/l; Molecular Probes, Carlsbad, CA, USA) for 15 min. After washing with PBS for three times, these cells were stained with PI (10 μmol/l; Molecular Probes) for 10 min. The living cells were observed using a 490 nm excitation filter, while the dead cells were observed using a 545 nm excitation filter.

### Immunofluoresence assay

After specific treatment, HLECs were fixed with ice‐cold methanol for 10 min. at −20°C. Cells were washed with PBS for three times. Non‐specific binding sites were blocked with 5% bovine serum albumin for 30 min. These cells were incubated with the primary antibody (Ki67, 1:200; Abcam, Cambridge, MA, USA) overnight at 4°C, and then incubated with the secondary antibody conjugated with Alexa Fluor 594 (Invitrogen) for 3 hrs at room temperature, followed by incubation with 4′,6‐Diamidino‐2‐phenylindole dihydrochloride (Sigma‐Aldrich, St. Louis, MO, USA) for 5 min. These cells were subsequently observed using the Olympus (DB80) microscope.

### MTT assay

Cell viability was detected using MTT assay. After specific treatment, 10 μl of 5 mg/ml MTT (in PBS) was added to each well of a 96‐well plate, and continually incubated at 37°C for 3 hrs. The formazan granules obtained in cells were then dissolved in dimethyl sulfoxide. The absorbance at 570 nm wavelength was detected using a microplate reader (Molecular Devices, Sunnyvale, CA, USA).

### Statistical analysis

All experiments were repeated at least three times. All data were expressed as mean ± S.E.M. Statistical significance was analyzed by Student's *t*‐test or one‐way anova followed by *post hoc* Bonferroni's test using SPSS 13.0 (SPSS Inc., Chicago, IL, USA). A *P* < 0.05 was considered statistically significant.

## Results

### Differential expression of lncRNAs between transparent and cataractous lenses

**Figure 1:**
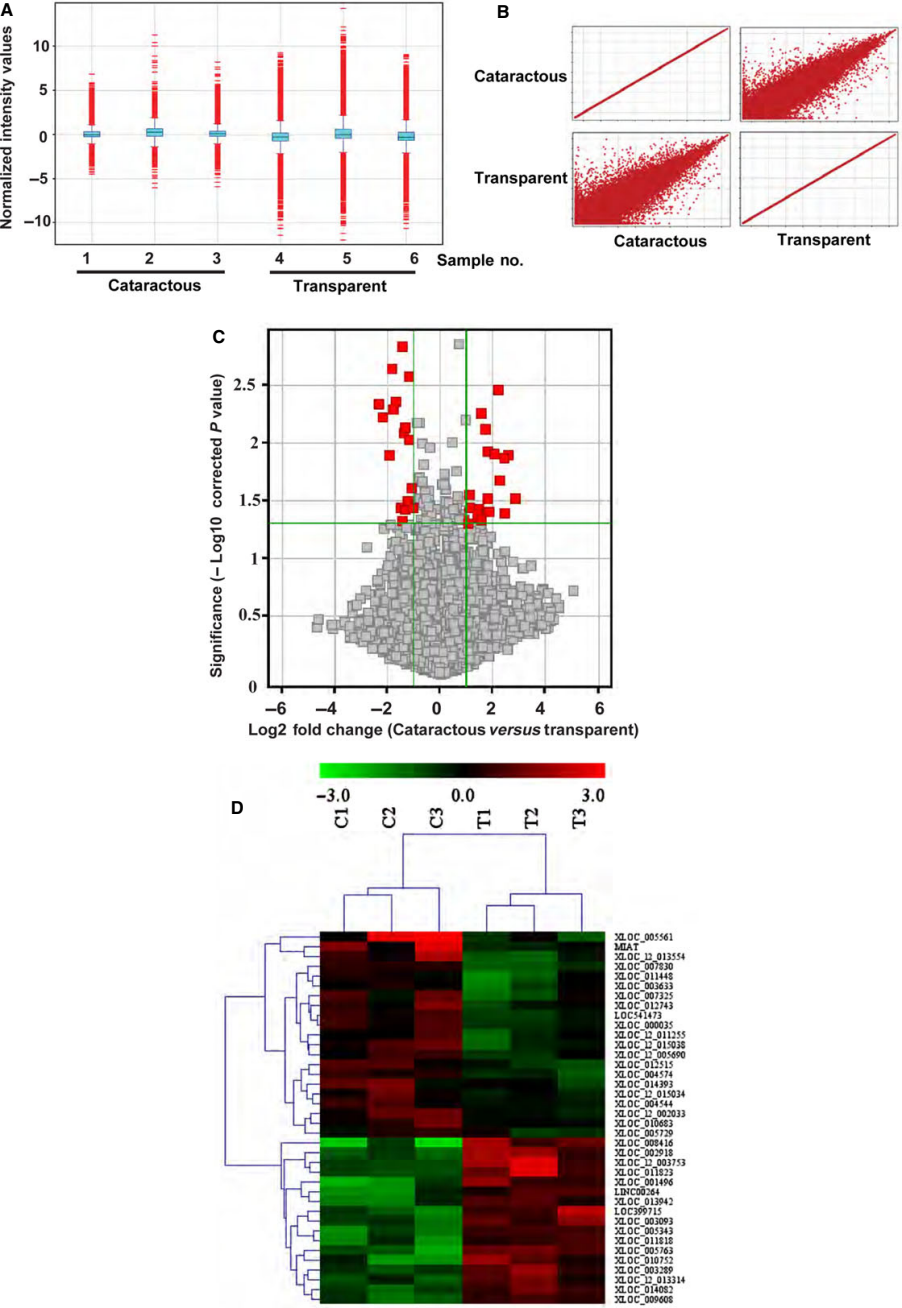
Differential expression of lncRNAs between transparent and cataractous lenses samples. (**A**) Box plot shows the distributions of lncRNA expression profiling. After normalization, the distributions of log 2 ratios among different samples are shown. The box plots consist of boxes with a central line and two tails. The central lines represent the median of the data, whereas the tails represent the upper and lower quartiles. (**B**) Scatter plot was used to compare the expression variations between transparent and cataractous lenses samples. (**C**) Volcano plot shows the differential expressed lncRNAs between transparent and cataractous lenses. (**D**) Heatmaps were generated from hierarchical cluster analysis to show differential expressed lncRNAs between transparent and cataractous lenses. The colour scale on the top illustrated the relative expression level of lncRNAs across different samples. Red denotes up‐regulation, while green denotes down‐regulation.

To identify cataract‐related lncRNAs, we extracted total RNAs from transparent and age‐matched cataractous lenses. The demographic and clinical features of study subjects for microarray analysis are shown in Table S1. Box plot demonstrates the difference between samples without making any assumption of statistical distribution. After normalization, the distributions of log 2 ratio between transparent and age‐matched cataractous lenses samples are shown in Figure 1A. Scatter plot demonstrates an overall indication of sample similarity between different groups. As shown in Figure 1B, the biological replicates had similar transcript levels (transparent *versus* transparent, cataractous *versus* cataractous), whereas a significant lncRNA expression difference was detected between transparent and cataractous lens samples.

The microarray data were filtered using volcano plot to show the differentially expressed lncRNAs between transparent and cataractous lens (Fig. 1C). We set a threshold as the fold change greater than 2.0. We identified 38 differentially expressed lncRNAs, including 17 down‐regulated lncRNAs and 21 up‐regulated lncRNAs (cataractous *versus* transparent; Table S2). To verify the results of microarray data, we performed quantitative RT‐PCRs (qRT‐PCRs), and found that 17 of 20 lncRNAs were verified to be differentially expressed between cataractous and transparent lens (Table 1). Among these lncRNAs, the greatest change was detected in lncRNA‐MIAT, which differed about nine‐fold between cataractous lens and transparent lens. This prompted us to explore the possible role of lncRNA‐MIAT in cataract occurrence in the following study. We also conducted hierarchical clustering analysis to obtain a systematic comparison of lncRNA expression between cataractous and transparent group. The cataractous samples were clustered together into the same branch, whereas the transparent samples were clustered into the other branch (Fig. 1D).

**Table 1:** Verification of microarray data using qRT‐PCRs

LncRNA name	Cataractous *versus* transparent			
	Microarray		qRT‐PCR	
XLOC_005561	7.63	Up	5.45	Up
XLOC_008416	5.97	Down	4.54	Up
MIAT	4.74	Up	9.19	Up
XLOC_l2_013554	4.73	Up	6.34	Up
XLOC_l2_003753	4.09	Down	5.62	Down
XLOC_002918	3.70	Down	4.33	Down
XLOC_001496	3.64	Down	2.10	Down
XLOC_007830	3.63	Up	3.76	Up
XLOC_005763	3.53	Down	2.99	Down
XLOC_011823	3.49	Down	3.22	Down
XLOC_007325	3.44	Up	4.42	Up
LOC399715	3.35	Down	2.43	Down
XLOC_010752	3.34	Down	3.23	Down
LOC541473	3.09	Up	5.62	Up
XLOC_004574	3.06	Up	4.77	Up
XLOC_011448	3.06	Up	3.45	Up
XLOC_000035	3.05	Up	4.21	Up

### Circulating MIAT level is up‐regulated in patients with cataract

**Figure 2:**
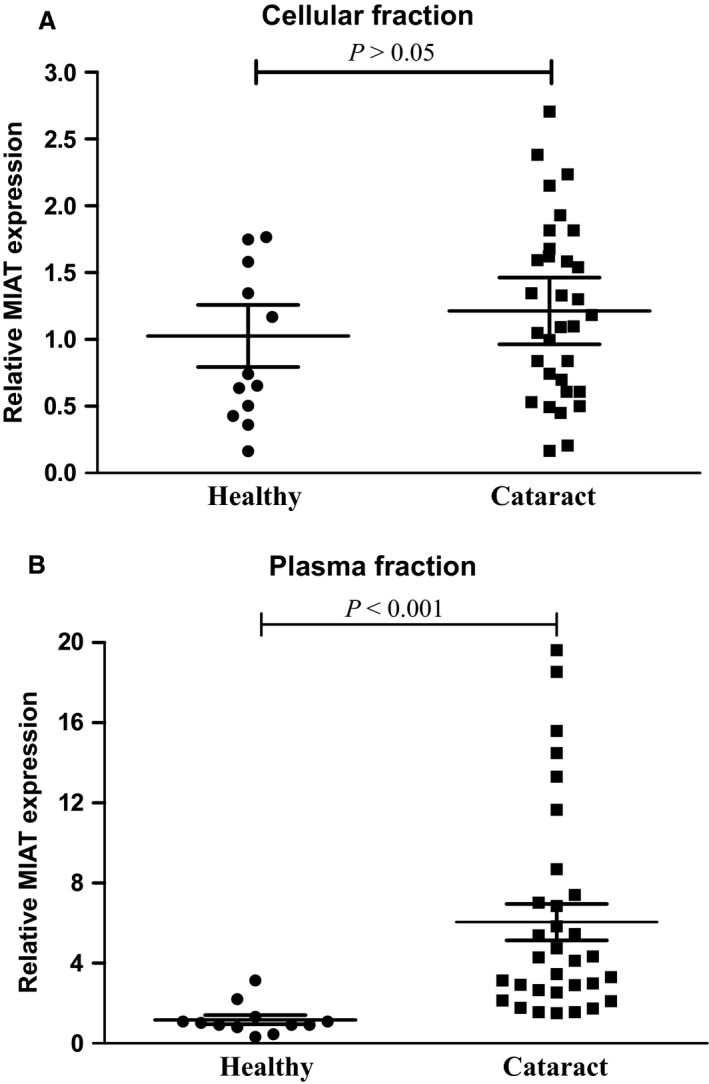
Circulating MIAT level is up‐regulated in patients with cataract. (**A** and **B**) The whole blood was collected from patients with cataract (*n* = 32) and age‐matched control (free of ocular diseases, *n* = 12). The whole blood was centrifuged to obtain the cellular fraction and plasma fraction.qRT‐PCRs were conducted to detect MIAT levels in cellular fraction (**A**) and plasma fraction (**B**). The data were expressed as relative change compared with the control group. Statistical significance was analyzed using anova *post hoc* Bonferroni's test.

As MIAT is up‐regulated in the pathological tissue of cataract patients, we have been suggested that its circulating level was also up‐regulated in patients with cataract. The demographic and clinical features of cataract patients and age‐matched controls for whole blood collection are shown in Table S3. We found that MIAT level was significantly up‐regulated in the plasma fraction but not the cellular fraction compared with that of the corresponding controls (Fig. 2A and B).

### LncRNA‐MIAT is shown as a cataract‐specific biomarker

**Figure 3:**
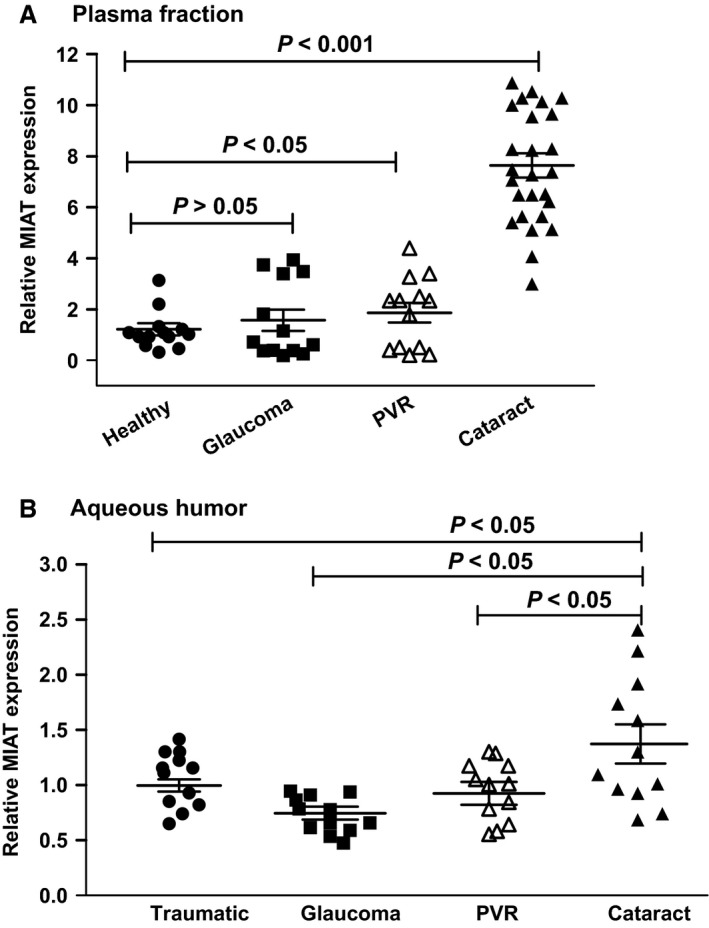
LncRNA‐MIAT is shown as a cataract‐specific biomarker. (**A**) Total RNAs were extracted from plasma fraction of whole blood collected from patients with cataract (*n* = 26), glaucoma (*n* = 13), PVR (*n* = 13) and healthy controls (*n* = 12). qRT‐PCRs were conducted to detect MIAT levels. The data were expressed as relative change compared with the healthy controls. (**B**) Total RNAs were extracted from the AH collected from patients with cataract (*n* = 12), patients with glaucoma (*n* = 12), patients with PVR (*n* = 12), and traumatic patients (*n* = 12, without cataract, glaucoma or PVR diseases). qRT‐PCRs were conducted to detect MIAT levels. Statistical significance was analyzed using anova *post hoc* Bonferroni's test.

To determine whether circulating MIAT is specifically up‐regulated in patients with cataract, we collected whole blood from cataract patients, PVR patients, glaucoma patients, and age‐matched controls (Table S4). MIAT level was significantly up‐regulated in the plasma fraction of cataract patients, but not in other individuals (Fig. 3A). Aqueous humor is one of the important body fluids in the eye, which is known to be related with various ocular diseases. We also detected MIAT expression pattern in the AH (Table S5). MIAT level was significantly up‐regulated in the AH of cataract patients, but not in other patients with glaucoma, PVR, or trauma (Fig. 3B). Collectively, these results suggest that MIAT is a cataract‐specific biomarker.

### Effects of MIAT knockdown on HLECs function

**Figure 4:**
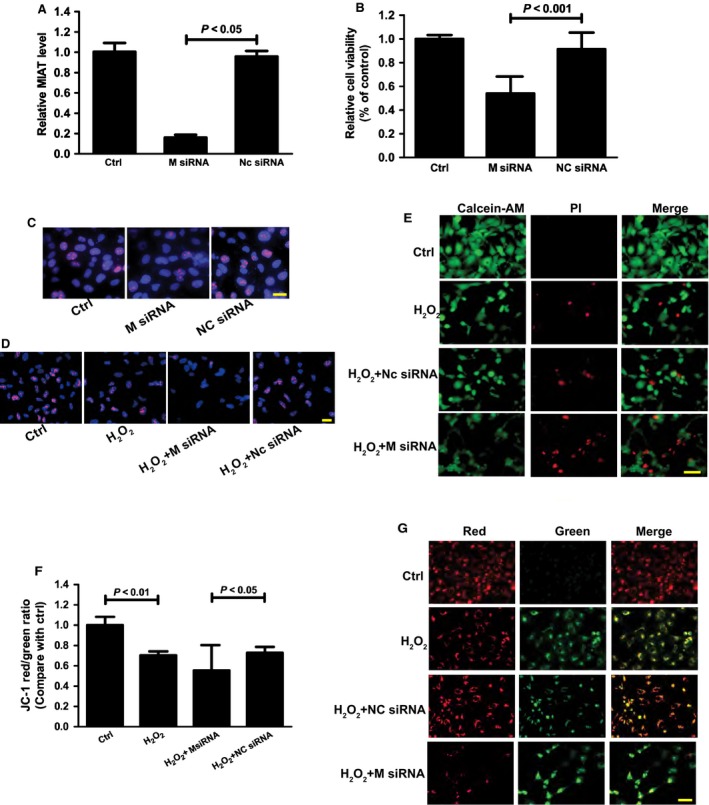
Effects of MIAT knockdown on HLECs function. (**A**–**C**) HLECs were transfected with MIAT siRNA, scramble siRNA (NC), or left untreated for 24 hrs. qRT‐PCRs were conducted to detected MIAT levels (**A**). Cell viability was detected using MTT method (*n* = 4) (**B**). Cell proliferation was detected using Ki67 staining (*n* = 3) (**C**); scale bar: 20 μm. (**D**–**G**) HLECs were transfected with MIAT siRNA, scramble siRNA (NC), or left untreated for 24 hrs, and then these cells were exposed to H_2_O_2_ (50 μM) for additional 48 hrs. Cell proliferation was detected using Ki67 staining (*n* = 3) (**D**); scale bar: 20 μm. Apoptotic cells were analyzed using calcein‐AM and PI double staining (*n* = 3) (**E**). Green: live cells, Red: dead or dying cell; scale bar: 50 μm. (**F** and **G**) HLECs were incubated with JC‐1 probe at 37 °C for 30 min., centrifuged, washed, transferred to a 96‐well plate (100,000 cells per well), assayed using a fluorescence plate reader (**F**), and observed using a fluorescence microscope (**G**). *n* = 3, scale bar: 50 μm. Statistical significance was analyzed using anova *post hoc* Bonferroni's test.

The LECs maintain normal physiology and homeostasis of the lens. Their dysfunction may lead to cataract formation 17. To determine whether MIAT regulates HLECs function *in vitro*, MIAT siRNA was transfected into HLECs to down‐regulate MIAT levels (Fig. 4A). MTT assay indicated that MIAT knockdown significantly reduced the viability of HLECs (Fig. 4B). Ki67 staining showed that MIAT knockdown significantly reduced the proliferation of HLECs (Fig. 4C). Oxidative stress is implicated in the process of cataract formation. Oxidative stimulation induced by hydrogen peroxide (H_2_O_2_, 50 μm) was performed to observe the effect of MIAT knockdown on HLECs proliferation under oxidative stress. Ki67 staining revealed that H_2_O_2_ (50 μM) treatment significantly decreased the proliferation of HLECs, whereas MIAT knockdown further reduced the proliferation of HLECs (Fig. 4D). Propidium iodide/Calcein‐AM staining revealed that H_2_O_2_ (50 μM) treatment significantly increased the number of dead or dying cells (as shown in red colour). MIAT siRNA but not scrambled siRNA transfection further increased the number of dead or dying cells (Fig. 4E). We also employed JC‐1 staining to detect the change in mitochondrial membrane potential, an indicator of cells' health and functional status. Compared with the group only treated with H_2_O_2_ (50 μM), MIAT knockdown resulted in a lower mitochondrial depolarization. MIAT knockdown also accelerated the shift of fluorescence emission from green to red (Fig. 4F and G).

### Effects of MIAT knockdown on posterior capsule opacification‐related pathological process

**Figure 5:**
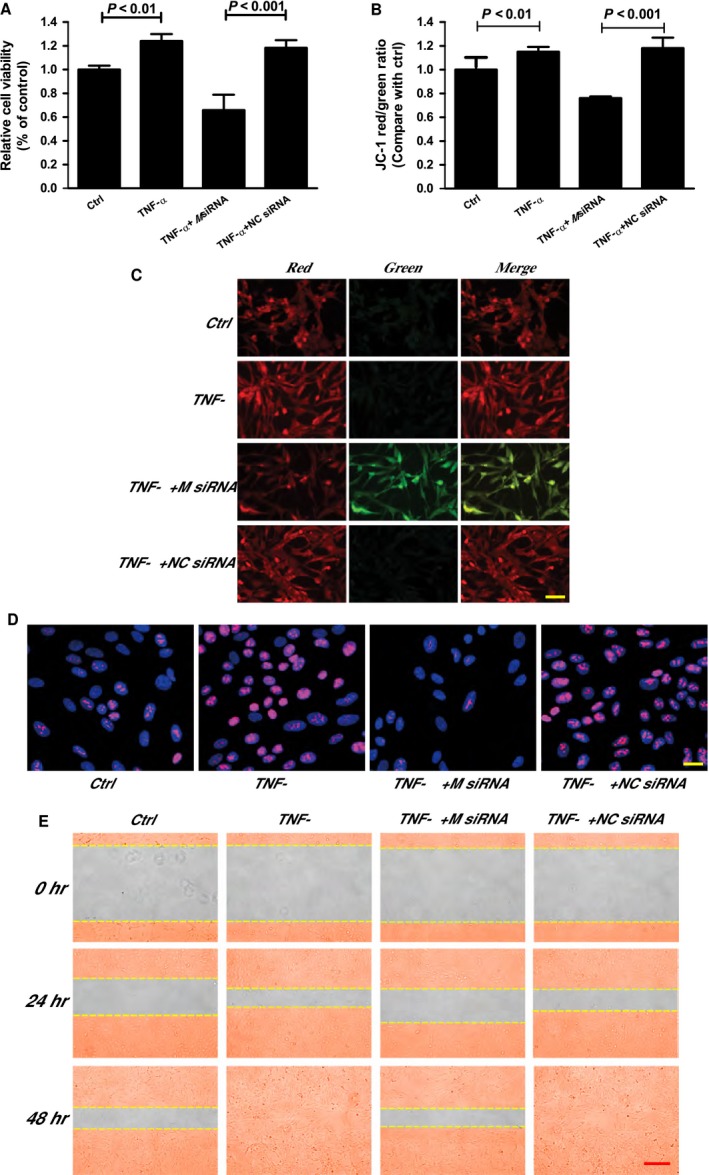
Effects of MIAT knockdown on PCO‐related pathological process. (**A**–**D**) HLECs were transfected with MIAT siRNA, scramble siRNA (NC), and then treated with TNF‐α (10 ng/ml) for 48 hrs. Cell viability was detected using MTT assay (*n* = 3) (**A**). HLECs were incubated with JC‐1 probe at 37°C for 30 min., centrifuged, washed, transferred to a 96‐well plate (100,000 cells per well), assayed using a fluorescence plate reader (**B**), and observed using a fluorescence microscope (**C**). *n* = 3, scale bar: 50 μm. (**E**) Cell proliferation was detected using Ki67 staining (*n* = 3), scale bar: 20 μm. (**D**). Cell migration was assessed using a wound‐healing assay. Images of wounded monolayer were taken at times 0, 24 and 48 hrs after treatment with TNF‐α. The horizontal lines indicated the wound edge. *n* = 3, scale bar: 100 μm. Statistical significance was analyzed using anova *post hoc* Bonferroni's test.

Posterior capsule opacification is a debilitating and relatively common complication of cataract surgery. The pathophysiology of this condition is tightly associated with abnormal production of cytokines, such as TNF‐α, interleukin (IL)‐1, IL‐6, IL‐8, Fibroblast growth factor (FGF), and transforming growth factor‐β. Their production could affect the proliferation and migration of HLECs, which in turn affects the remodeling of lens capsule and cataract formation 18, 19. We found that TNF‐α treatment resulted in a significant increase in the viability of HLECs. MIAT knockdown could significantly reduce this increase (Fig. 5A). Ki67 staining revealed that TNF‐α treatment led to a significant increase in the proliferation ability of HLECs, whereas MIAT knockdown could reduce this proliferation (Fig. 5B). JC‐1 staining showed that MIAT knockdown cells exhibited a marked decrease in red fluorescence (polarized state) and a significant increase in green fluorescence (depolarized state; Fig. 5C and D). Moreover, we found that MIAT knockdown could decrease the number of migrated cells‐induced by TNF‐α treatment (Fig. 5E). Taken together, these results show that MIAT regulates the proliferation and migration of HLECs involved in PCO‐related pathological process.

### MIAT/miR‐150‐5p/Akt is involved in the regulation of HLEC function

**Figure 6:**
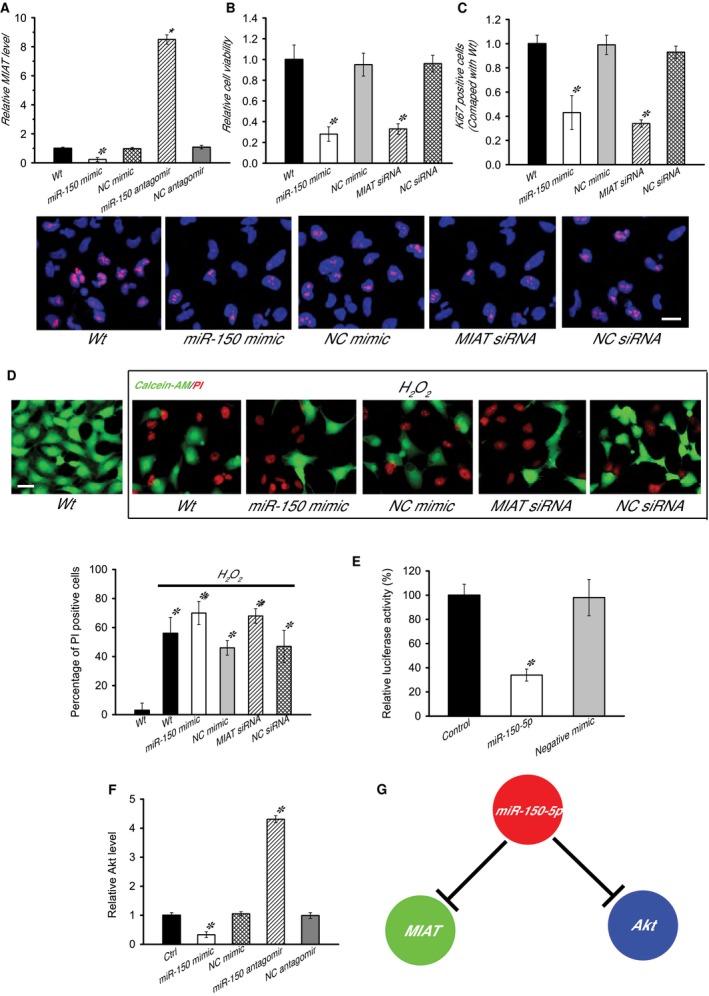
MIAT/miR‐150‐5p/Akt is involved in the regulation of HLEC function. (**A**) HLECs were transfected with miR‐150‐5p mimic, NC mimic, miR‐150‐5p antagomir, NC antagomir, or left untreated. MIAT levels were detected using qRT‐PCRs, and expressed as relative change compared with wild‐type (Wt) group (*n* = 3). (**B** and **C**) HLECs were treated as shown. MTT assay was performed to detect cell viability. The data were expressed as relative change compared with Wt group (*n* = 6). Data were shown as mean ± S.E.M. A representative image was shown for Ki67 staining of HLECs along with the quantification of Ki67 positive cells (*n* = 3), scale bar: 20 μm. (**D**) Apoptotic cells were analyzed using calcein‐AM and PI double staining (*n* = 3). Green: live cells, Red: dead or dying cell, scale bar: 50 μm. (**E**) Akt 3′UTR was cloned into the downstream of luciferase vector (RLuc‐Akt‐Wt) and transfected with miR‐150‐5p mimic or negative mimic. Luciferase activity was detected using dual luciferase assay (*n* = 4). The data were expressed as relative control compared with the RLuc‐Akt‐Wt alone group. (**F**) HLECs were transfected with miR‐150‐5p mimic, NC mimic, miR‐150‐5p antagomir, NC antagomir or left untreated. Akt levels were detected using qRT‐PCRs, and expressed as relative change compared with Wt group (*n* = 4). (**G**) A diagram showed that potential interaction among MIAT, miR‐150‐5p, and Akt. ‘*’ indicated a significant difference compared with the control or Wt group. ‘#’ indicated a significant difference compared with H_2_O_2_ treatment group. Statistical significance was analyzed using anova *post hoc* Bonferroni's test.

In our previous study, we found that LncRNA‐MIAT could act as miRNA‐150‐5p sponge, and regulate miRNA‐150‐5p available for binding its target gene 20. We thus investigated whether miRNA‐150‐5p regulates MIAT level in HLECs. miR‐150‐5p mimic injection resulted in a marked reduction in MIAT level. By contrast, miR‐150‐5p antagomir injection significantly up‐regulated MIAT level in HLECs. This result suggested that miR‐150‐5p directly regulated MIAT level in HLECs (Fig. 6A).

To reveal the role of miR‐150‐5p in HLEC function, HLECs were transfected with miR‐150‐5p mimic to up‐regulate its level. miR‐150‐5p mimic transfection significantly decreased HLEC viability (Fig. 6B), inhibited the proliferation ability of HLECs (Fig. 6C). In response to oxidative stress (H_2_O_2_, 50 μM), miR‐150‐5p mimic transfection could accelerate the development of HLECs apoptosis (Fig. 6D). Collectively, these results suggest that miR‐150‐5p is a key regulator of HLEC function.

Among the putative targets of miR‐150‐5p, we focused on Akt (with three miR‐150‐5p sites), a key regulator involved in regulating cell proliferation. The 3′‐UTR of Akt was fused to the luciferase coding region (RLuc‐Akt‐WT) and transfected into HLECs with miR‐150‐5p mimic in parallel to the negative control. Luciferase assay showed that Akt was a target of miR‐150‐5p (Fig. 6E). miR‐150‐5p mimic injection resulted in a marked reduction in Akt level. By contrast, miR‐150‐5p antagomir injection significantly up‐regulated Akt level in HLECs (Fig. 6F).

We further determined whether MIAT‐Akt crosstalk is involved in the regulation of HLEC function (Fig. 6G). MTT assay revealed that MIAT knockdown significantly decreased the HLEC viability, whereas exogenous Akt activator could reverse this decrease in cell viability (Fig. S1). Ki67 staining suggested that MIAT knockdown obviously inhibited HLEC proliferative ability, whereas exogenous Akt activator could promote HLEC proliferation (Fig. S1). In response to oxidative stress, exogenous Akt activator treatment could partially prevent MIAT knockdown‐mediated HLEC apoptosis (Fig. S1). Taken together, these results suggest that MIAT‐Akt crosstalk is involved in the regulation of HLEC function.

## Discussion

LncRNAs have emerged as novel regulators of many biological processes and human disorders 11, 21. However, their roles in age‐related cataract are still unclear. Here, we identified 38 differentially expressed lncRNAs between cataractous and transparent lens. Of them, lncRNA‐MIAT level was up‐regulated in the lens, AH, and plasma fraction of whole blood in cataract patients. *In vitro* study revealed the important role of lncRNA‐MIAT in regulating HLECs proliferation and migration.

Circulating nucleic acids (CNAs) have received increasing attention because of the application as a non‐invasive, rapid and sensitive tool for molecular diagnosis of human disorders 22, 23. Circulating levels of some miRNAs, lncRNA‐MALAT1 or lncRNA‐Vax2os1/Vax2os2 were altered in the AH in several ocular diseases, including cataract, diabetic retinopathy and age‐related macular degeneration 13, 14, 24. Here, we found that lncRNA‐MIAT level was specifically up‐regulated in the plasma fraction of cataract patients. Thus, lncRNA‐MIAT detection may provide a novel method for molecular diagnosis or monitoring of lenticular diseases.

Although CNA circulates freely in blood plasma both in disease and in health, the source still remains enigmatic. In a healthy person, CNA may enter circulation *via* the apoptosis of lymphocytes and other nucleated cells. Apoptosis as the primary source of CNA has been confirmed in many cancer researches 25. Lens epithelial cell apoptosis is an early event during cataract formation 26. We speculated MIAT release in LECs may enter into circulation *via* some specific ways 23, 27. It is well known that RNA is very labile and easily degraded by ubiquitously present RNases. We speculated the presence of stable endogenous or exogenous circulating lncRNA‐MIAT in blood may be mediated by some specific mechanisms, such as (*i*) lncRNA may contain in apoptotic bodies; or (*ii*) RNA bound to proteins/phospholipids and was protected from degradation by nucleases 25. In addition, circulating lncRNA‐MIAT raises the question that whether it has a physiological and/or pathological role or whether it functions in a manner similar to hormones. We speculated that circulating lncRNA‐MIAT may take part in cell‐to‐cell communication and signaling in normal biology and in pathophysiology during cataract formation. The field of extracellular nucleic acids is still in its infancy. Further studies are still required for addressing the above‐mentioned questions.

Aqueous humor supplies nutrients and removes the metabolic waste from the avascular tissues of the eye such as lens and cornea 28. Change in AH protein content is associated with cataract formation. α and γ crystallin content in AH is significantly different between cataractous and transparent lens 29. MIAT level was significantly up‐regulated in the AH of cataract patients. By contrast, MIAT level in AH collected from patients with known ocular diseases other than cataract did not increase. We speculated that the increase in local MIAT level may be secreted by diseased lens cells. MIAT presence in AH suggests that MIAT may have functional roles in regulating target genes in tissues lining the anterior chamber. MIAT increase may change gene regulatory network involved in cataract formation.

Age‐related cataract is a cause of blindness involving genetic and environmental influences 30. Lens cells are constantly exposed to oxidative stress from reactive oxygen species such as H_2_O_2_, hypochlorous acid (HClO), the free radicals superoxide (O_2_˙^−^) and hydroxyl radical (˙OH), suggesting that oxidative stress is involved in the etiology of age‐related cataract 3, 31. The patients with cataract usually have the accumulated products of oxidative stress. Correspondingly, they have higher MIAT expression than the matched controls. Oxidative stress reduces the viability and proliferation ability of HLECs, whereas MIAT knockdown further decreases the viability and proliferation ability of HLECs, implying increased MIAT level is a compensatory response to combat against oxidative stress.

Posterior capsule opacification is the main complication of cataract surgery. Following the insult of surgery, residual LECs rapidly grow at the equator and under the anterior lens capsule. These cells proliferate and migrate onto the posterior capsule 32, 33. The response of LECs can be considered a wound‐healing reaction resulting from the activation of inflammatory cells and production of cytokines and growth factors after surgery 34. We found that MIAT knockdown inhibits TNF‐α‐induced proliferation and migration of HLECs, suggesting that MIAT intervention could affect PCO formation.

In this study, we show that 38 lncRNAs are differentially expressed between transparent and cataractous lenses. LncRNA‐MIAT is specifically up‐regulated in the plasma fraction of whole blood or AH of cataract patients. MIAT knockdown could regulate HLECs function upon oxidative stress and inflammatory factor stimulus. This study provides a novel insight into the pathogenesis of age‐related cataract.

## Conflicts of interest

The authors confirm that there are no conflicts of interest.

## Supporting information


**Figure S1** MIAT‐Akt crosstalk is involved in the regulation of HLEC function. (A and B) HLECs were transfected with MIAT siRNA or scramble siRNA, and with or without Akt overexpression for 48 hrs. Cell viability was detected using MTT assay (A). Cell proliferation was determined using Ki67 staining and quantified (B). (C) HLECs were transfected as shown and exposed to H_2_O_2_ (50 μM) for 48 hrs. Apoptotic cells were observed by PI staining. Data were shown as mean ± S.E.M., and expressed as relative change compared with the control group (*n* = 4, *P < 0.05). ‘*’ indicated a significant difference compared with the corresponding control group. ‘#’ indicated a significant difference between the marked groups.

**Table S1** Demographic and clinical features of study subjects for microarray analysis.

**Table S2** Differential expressed lncRNAs between transparent lenses and cataractous lenses.

**Table S3** Demographic and clinical features of study subjects for circulating MIAT detection.

**Table S4** Demographic and clinical features of study subjects for peripheral blood collection.

**Table S5** Demographic and clinical features of study subjects for AH collection.
