# Supplementary material for: Role of long non‐coding RNA MIAT in proliferation, apoptosis and migration of lens epithelial cells: a clinical and in vitro study
Source: J Cell Mol Med. 2016 Jan 28;20(3):537–48. doi: 10.1111/jcmm.12755 (PMC4759467; doi:10.1111/jcmm.12755)
Supplement: Supplementary file 2 — Table S1 Demographic and clinical features of study subjects for microarray analysis. [file JCMM-20-537-s002.doc]

**Table S1: Demographic and clinical features of study subjects for microarray analysis**

| **Group No** | **Lenticular opacification** | **Age** | **Gender** |
| --- | --- | --- | --- |
| Cataract 1 | NO_6_NC_5_C_2_P_3_ | 66 | F |
| Cataract 2 | NO_6_NC_6_C_2_P_3_ | 65 | M |
| Cataract 3 | NO_5_NC_5_C_3_P_2_ | 60 | F |
| Cataract 4 | NO_4_NC_4_C_3_P_2_ | 58 | F |
| Cataract 5 | NO_5_NC_4_C_2_P_3_ | 64 | M |
| Cataract 6 | NO_6_NC_4_C_3_P_3_ | 70 | F |
| Cataract 7 | NO_6_NC_5_C_2_P_3_ | 56 | F |
| Cataract 8 | NO_5_NC_6_C_3_P_3_ | 68 | F |
| Cataract 9 | NO_5_NC_5_C_2_P_2_ | 49 | M |
| Control 1 | NO_2_NC_2_C_1_P_1_ | 65 | M |
| Control 2 | NO_2_NC_2_C_1_P_1_ | 58 | F |
| Control 3 | NO_1_NC_2_C_1_P_1_ | 63 | M |
| Control 4 | NO_1_NC_2_C_1_P_1_ | 55 | F |
| Control 5 | NO_2_NC_1_C_1_P_1_ | 60 | M |
| Control 6 | NO_2_NC_2_C_1_P_1_ | 69 | F |
| Control 7 | NO_2_NC_2_C_1_P_1_ | 57 | F |
| Control 8 | NO_2_NC_2_C_1_P_1_ | 64 | M |
| Control 9 | NO_2_NC_1_C_1_P_1_ | 54 | M |

Note: Lens Opacities Classification System III (LOCS III) was used to grade age-related cataract.
