# Supplementary material for: Role of long non‐coding RNA MIAT in proliferation, apoptosis and migration of lens epithelial cells: a clinical and in vitro study
Source: J Cell Mol Med. 2016 Jan 28;20(3):537–48. doi: 10.1111/jcmm.12755 (PMC4759467; doi:10.1111/jcmm.12755)
Supplement: Supplementary file 4 — Table S3 Demographic and clinical features of study subjects for circulating MIAT detection. [file JCMM-20-537-s004.doc]

**Table S3: Demographic and clinical features of study subjects** for circulating MIAT detection

| **Group No** | **Lenticular opacification** | **Age** | **Gender** |
| --- | --- | --- | --- |
| Cataract 1 | NO_6_NC_5_C_2_P_3_ | 66 | F |
| Cataract 2 | NO_6_NC_6_C_2_P_3_ | 65 | M |
| Cataract 3 | NO_5_NC_5_C_3_P_2_ | 60 | F |
| Cataract 4 | NO_4_NC_4_C_3_P_2_ | 58 | F |
| Cataract 5 | NO_5_NC_4_C_2_P_3_ | 64 | M |
| Cataract 6 | NO_6_NC_4_C_3_P_3_ | 70 | F |
| Cataract 7 | NO_6_NC_5_C_2_P_3_ | 56 | F |
| Cataract 8 | NO_5_NC_6_C_3_P_3_ | 68 | F |
| Cataract 9 | NO_5_NC_5_C_2_P_2_ | 49 | M |
| Cataract 10 | NO_4_NC_4_C_3_P_2_ | 59 | M |
| Cataract 11 | NO_5_NC_4_C_3_P_3_ | 50 | M |
| Cataract 12 | NO_6_NC_5_C_3_P_3_ | 61 | F |
| Cataract 13 | NO_5_NC_4_C_2_P_2_ | 53 | M |
| Cataract 14 | NO_6_NC_4_C_2_P_3_ | 57 | F |
| Cataract 15 | NO_5_NC_4_C_2_P_2_ | 51 | M |
| Cataract 16 | NO_5_NC_4_C_2_P_3_ | 56 | F |
| Cataract 17 | NO_5_NC_6_C_3_P_3_ | 53 | F |
| Cataract 18 | NO_5_NC_5_C_2_P_3_ | 58 | M |
| Cataract 19 | NO_4_NC_5_C_2_P_3_ | 54 | M |
| Cataract 20 | NO_5_NC_5_C_3_P_2_ | 55 | M |
| Cataract 21 | NO_6_NC_4_C_3_P_2_ | 54 | F |
| Cataract 22 | NO_5_NC_6_C_2_P_3_ | 61 | M |
| Cataract 23 | NO_4_NC_6_C_2_P_2_ | 48 | M |
| Cataract 24 | NO_5_NC_5_C_2_P_2_ | 61 | F |
| Cataract 25 | NO_5_NC_6_C_2_P_2_ | 51 | M |
| Cataract 26 | NO_5_NC_4_C_3_P_2_ | 62 | F |
| Cataract 27 | NO_4_NC_4_C_3_P_2_ | 52 | F |
| Cataract 28 | NO_5_NC_5_C_2_P_3_ | 60 | M |
| Cataract 29 | NO_5_NC_4_C_3_P_2_ | 59 | M |
| Cataract 30 | NO_4_NC_6_C_2_P_2_ | 50 | M |
| Cataract 31 | NO_5_NC_5_C_3_P_2_ | 61 | F |
| Cataract 32 | NO_5_NC_4_C_2_P_3_ | 53 | F |
| Control 1 | NO_2_NC_2_C_1_P_1_ | 61 | M |
| Control 2 | NO_2_NC_2_C_1_P_1_ | 49 | F |
| Control 3 | NO_1_NC_2_C_1_P_1_ | 56 | M |
| Control 4 | NO_1_NC_2_C_1_P_1_ | 47 | F |
| Control 5 | NO_2_NC_1_C_1_P_1_ | 58 | M |
| Control 6 | NO_2_NC_2_C_1_P_1_ | 51 | F |
| Control 7 | NO_2_NC_2_C2_1_P_1_ | 61 | F |
| Control 8 | NO_2_NC_2_C_1_P_1_ | 52 | M |
| Control 9 | NO_2_NC_1_C_1_P_2_ | 57 | M |
| Control 10 | NO_2_NC_2_C_1_P_1_ | 50 | F |
| Control 11 | NO_2_NC_2_C_1_P_1_ | 61 | F |
| Control 12 | NO_2_NC_2_C_2_P_1_ | 49 | M |

Note:

The patients having history of cancer, asthma, diabetes mellitus, cardiovascular diseases, and ocular diseases other than cataract were excluded.

The control group having history of cancer, asthma, diabetes mellitus, cardiovascular diseases, and ocular diseases were excluded.
