# Supplementary material for: Role of long non‐coding RNA MIAT in proliferation, apoptosis and migration of lens epithelial cells: a clinical and in vitro study
Source: J Cell Mol Med. 2016 Jan 28;20(3):537–48. doi: 10.1111/jcmm.12755 (PMC4759467; doi:10.1111/jcmm.12755)
Supplement: Supplementary file 5 — Table S4 Demographic and clinical features of study subjects for peripheral blood collection. [file JCMM-20-537-s005.doc]

**Table S4: Demographic and clinical features of study subjects for peripheral blood collection**

| **Group No** | **Lenticular opacification** | **Age** | **Gender** |
| --- | --- | --- | --- |
| Cataract 1 | NO_6_NC_5_C_2_P_3_ | 56 | F |
| Cataract 2 | NO_5_NC_6_C_3_P_3_ | 68 | F |
| Cataract 3 | NO_5_NC_5_C_2_P_2_ | 49 | M |
| Cataract 4 | NO_4_NC_4_C_3_P_2_ | 59 | M |
| Cataract 5 | NO_5_NC_4_C_3_P_3_ | 50 | M |
| Cataract 6 | NO_4_NC_5_C_3_P_3_ | 61 | F |
| Cataract 7 | NO_5_NC_4_C_2_P_2_ | 53 | M |
| Cataract 8 | NO_6_NC_4_C_2_P_3_ | 57 | F |
| Cataract 9 | NO_5_NC_4_C_2_P_2_ | 51 | M |
| Cataract 10 | NO_5_NC_4_C_2_P_3_ | 56 | F |
| Cataract 11 | NO_5_NC_6_C_3_P_3_ | 53 | F |
| Cataract 12 | NO_5_NC_5_C_2_P_3_ | 58 | M |
| Cataract 13 | NO_4_NC_5_C_2_P_3_ | 54 | M |
| Cataract 14 | NO_5_NC_5_C_3_P_2_ | 55 | M |
| Cataract 15 | NO_6_NC_4_C_3_P_2_ | 54 | F |
| Cataract 16 | NO_5_NC_6_C_2_P_3_ | 61 |  |
| Cataract 17 | NO_4_NC_6_C_2_P_2_ | 48 | M |
| Cataract 18 | NO_5_NC_5_C_2_P_2_ | 61 | F |
| Cataract 19 | NO_5_NC_6_C_2_P_2_ | 51 | M |
| Cataract 20 | NO_6_NC_4_C_3_P_2_ | 62 | F |
| Cataract 21 | NO_4_NC_4_C_3_P_2_ | 52 | F |
| Cataract 22 | NO_5_NC_5_C_2_P_3_ | 60 | M |
| Cataract 23 | NO_5_NC_4_C_3_P_2_ | 59 | M |
| Cataract 24 | NO_4_NC_6_C_2_P_2_ | 50 | M |
| Cataract 25 | NO_5_NC_5_C_3_P_2_ | 61 | F |
| Cataract 26 | NO_5_NC_4_C_2_P_3_ | 53 | F |
| Control 1 | NO_2_NC_2_C_1_P_1_ | 59 | F |
| Control 2 | NO_2_NC_2_C_1_P_1_ | 52 | F |
| Control 3 | NO_2_NC_2_C_1_P_1_ | 56 | M |
| Control 4 | NO_2_NC_2_C_1_P_1_ | 49 | F |
| Control 5 | NO_2_NC_2_C_1_P_1_ | 56 | M |
| Control 6 | NO_2_NC_2_C_2_P_1_ | 55 | M |
| Control 7 | NO_2_NC_2_C_1_P_1_ | 63 | F |
| Control 8 | NO_1_NC_2_C_1_P_2_ | 54 | M |
| Control 9 | NO_2_NC_1_C_1_P_1_ | 57 | F |
| Control 10 | NO_2_NC_2_C_1_P_1_ | 53 | F |
| Control 11 | NO_1_NC_2_C_1_P_1_ | 63 | M |
| Control 12 | NO_2_NC_2_C_2_P_1_ | 55 | M |
| PVR 1 | NO_2_NC_2_C_1_P_1_ | 61 | M |
| PVR 2 | NO_2_NC_2_C_1_P_1_ | 52 | M |
| PVR 3 | NO_2_NC_2_C_1_P_1_ | 57 | F |
| PVR 4 | NO_1_NC_2_C_1_P_1_ | 49 | F |
| PVR 5 | NO_1_NC_2_C_1_P_1_ | 59 | F |
| PVR 6 | NO_2_NC_1_C_1_P_1_ | 48 | M |
| PVR 7 | NO_2_NC_2_C_1_P_1_ | 62 | M |
| PVR 8 | NO_2_NC_2_C_1_P_1_ | 52 | F |
| PVR 9 | NO_2_NC_2_C_1_P_1_ | 57 | M |
| PVR 10 | NO_2_NC_1_C_1_P_1_ | 53 | M |
| PVR 11 | NO_2_NC_2_C_1_P_1_ | 55 | F |
| PVR 12 | NO_2_NC_2_C_1_P_1_ | 49 | F |
| PVR 13 | NO_2_NC_2_C_1_P_1_ | 64 | M |
| Glaucoma 1 | NO_2_NC_2_C_2_P_1_ | 59 | F |
| Glaucoma 2 | NO_2_NC_2_C_1_P_1_ | 53 | F |
| Glaucoma 3 | NO_1_NC_2_C_1_P_1_ | 57 | M |
| Glaucoma 4 | NO_1_NC_2_C_1_P_1_ | 54 | F |
| Glaucoma 5 | NO_2_NC_1_C_2_P_1_ | 56 | M |
| Glaucoma 6 | NO_2_NC_2_C_2_P_1_ | 54 | M |
| Glaucoma 7 | NO_2_NC_2_C_1_P_2_ | 51 | M |
| Glaucoma 8 | NO_2_NC_2_C_1_P_2_ | 52 | F |
| Glaucoma 9 | NO_1_NC_2_C_1_P_1_ | 62 | M |
| Glaucoma 10 | NO_2_NC_2_C_1_P_1_ | 49 | F |
| Glaucoma 11 | NO_2_NC_2_C_1_P_1_ | 60 | M |
| Glaucoma 12 | NO_2_NC_2_C_1_P_1_ | 53 | M |
| Glaucoma 13 | NO_1_NC_2_C_2_P_1_ | 61 | F |

Note: The patients having history of cancer, asthma, diabetes mellitus, cardiovascular diseases, and ocular diseases other than glaucoma, cataract, and PVR were excluded. The control group having history of cancer, asthma, diabetes mellitus, cardiovascular diseases, and ocular diseases were excluded.
