# Supplementary material for: Role of long non‐coding RNA MIAT in proliferation, apoptosis and migration of lens epithelial cells: a clinical and in vitro study
Source: J Cell Mol Med. 2016 Jan 28;20(3):537–48. doi: 10.1111/jcmm.12755 (PMC4759467; doi:10.1111/jcmm.12755)
Supplement: Supplementary file 6 — Table S5 Demographic and clinical features of study subjects for AH collection. [file JCMM-20-537-s006.doc]

**Table S5: Demographic and clinical features of study subjects for AH collection**

| **Group No** | **Lenticular opacification** | **Age** | **Gender** |
| --- | --- | --- | --- |
| Cataract 1 | NO_6_NC_5_C_2_P_3_ | 61 | F |
| Cataract 2 | NO_5_NC_6_C_3_P_3_ | 47 | F |
| Cataract 3 | NO_5_NC_5_C_2_P_2_ | 57 | M |
| Cataract 4 | NO_4_NC_4_C_3_P_2_ | 52 | M |
| Cataract 5 | NO_5_NC_4_C_3_P_3_ | 57 | M |
| Cataract 6 | NO_6_NC_5_C_3_P_3_ | 47 | F |
| Cataract 7 | NO_5_NC_4_C_2_P_2_ | 62 | M |
| Cataract 8 | NO_6_NC_4_C_2_P_3_ | 50 | M |
| Cataract 9 | NO_5_NC_4_C_2_P_2_ | 56 | M |
| Cataract 10 | NO_5_NC_4_C_2_P_3_ | 53 | F |
| Cataract 11 | NO_5_NC_6_C_3_P_3_ | 60 | F |
| Cataract 12 | NO_5_NC_5_C_2_P_3_ | 51 | M |
| Trauma 1 | NO_2_NC_2_C_1_P_1_ | 59 | M |
| Trauma 2 | NO_2_NC_2_C_1_P_1_ | 53 | F |
| Trauma 3 | NO_1_NC_2_C_1_P_1_ | 61 | M |
| Trauma 4 | NO_1_NC_2_C_1_P_1_ | 53 | F |
| Trauma 5 | NO_2_NC_1_C_1_P_1_ | 56 | F |
| Trauma 6 | NO_2_NC_2_C_1_P_1_ | 54 | M |
| Trauma 7 | NO_2_NC_2_C_1_P_1_ | 58 | M |
| Trauma 8 | NO_2_NC_2_C_1_P_1_ | 55 | F |
| Trauma 9 | NO_2_NC_1_C_1_P_1_ | 54 | M |
| Trauma 10 | NO_2_NC_2_C_1_P_1_ | 60 | M |
| Trauma 11 | NO_2_NC_2_C_1_P_1_ | 52 | F |
| Trauma 12 | NO_2_NC_2_C_2_P_1_ | 62 | F |
| PVR 1 | NO_2_NC_2_C_2_P_1_ | 58 | F |
| PVR 2 | NO_2_NC_2_C_1_P_1_ | 61 | M |
| PVR 3 | NO_2_NC_2_C_1_P_1_ | 49 | F |
| PVR 4 | NO_1_NC_2_C_1_P_1_ | 58 | F |
| PVR 5 | NO_1_NC_2_C_1_P_1_ | 67 | F |
| PVR 6 | NO_2_NC_1_C_2_P_1_ | 56 | M |
| PVR 7 | NO_2_NC_2_C_1_P_1_ | 59 | M |
| PVR 8 | NO_2_NC_2_C_1_P_1_ | 56 | F |
| PVR 9 | NO_2_NC_2_C_1_P_1_ | 54 | M |
| PVR 10 | NO_2_NC_1_C_1_P_1_ | 61 | M |
| PVR 11 | NO_2_NC_2_C_1_P_1_ | 67 | F |
| PVR 12 | NO_2_NC_2_C_1_P_1_ | 55 | M |
| Glaucoma 1 | NO_2_NC_2_C_1_P_1_ | 62 | F |
| Glaucoma 2 | NO_2_NC_2_C_1_P_1_ | 62 | F |
| Glaucoma 3 | NO_1_NC_2_C_1_P_1_ | 55 | M |
| Glaucoma 4 | NO_1_NC_2_C_1_P_1_ | 47 | F |
| Glaucoma 5 | NO_2_NC_1_C_2_P_1_ | 62 | F |
| Glaucoma 6 | NO_2_NC_2_C_1_P_1_ | 64 | F |
| Glaucoma 7 | NO_2_NC_2_C_1_P_1_ | 55 | M |
| Glaucoma 8 | NO_2_NC_2_C_1_P_1_ | 57 | M |
| Glaucoma 9 | NO_1_NC_2_C_1_P_1_ | 56 | M |
| Glaucoma 10 | NO_2_NC_2_C_1_P_1_ | 68 | M |
| Glaucoma 11 | NO_2_NC_2_C_1_P_1_ | 65 | F |
| Glaucoma 12 | NO_2_NC_2_C_1_P_1_ | 53 | M |

Note: The patients having history of cancer, asthma, diabetes mellitus, cardiovascular diseases, and ocular diseases other than glaucoma, cataract, and PVR were excluded. The control group was traumatic patients having no history of cancer, asthma, diabetes mellitus, cardiovascular diseases, and ocular diseases.
